# Building Families’ Capacities: Community Forums with Parents and Occupational Therapists

**DOI:** 10.1177/00084174231160972

**Published:** 2023-05-15

**Authors:** Marie Grandisson, Sarah Martin-Roy, Justine Marcotte, Élise Milot, Rébecca Girard, Emmanuelle Jasmin, Cynthia Fauteux, Julie Bergeron

**Keywords:** Occupational therapy, Children with special needs, Children with disability, Family-centered, Capacity building, Approche axée sur la famille, enfants avec besoins particuliers, enfants ayant un handicap, ergothérapie, renforcement des capacités

## Abstract

**Background.** Parents of a child considered to have special needs are at greater risk of stress and exhaustion. Although many occupational therapy interventions can help these children, they often require significant time and energy from families. **Purpose.** To document the perspectives of parents and occupational therapists regarding ways to offer services that help build families’ capacities without overloading them. **Method.** A qualitative descriptive design guided online community forums with 41 parents and occupational therapists in Quebec, Canada. **Findings.** Nine key principles to build the capacities of families without overburdening them were identified. These include being sensitive to possible negative impacts of services, avoiding overwhelming the family with information or recommendations, taking the needed time, highlighting the positive, and offering flexible conditions for services. **Implications.** Our findings help identify how capacity-building rehabilitation services can be offered to families to optimize positive outcomes and minimize harms.

## Résumé

**Description.** Les parents d’enfants considérés comme ayant des besoins particuliers sont plus vulnérables au stress et à l’épuisement. Si de nombreuses interventions d’ergothérapie peuvent aider ces enfants, celles-ci nécessitent souvent beaucoup de temps et d’énergie de la part des familles. **But.** Documenter les perspectives des parents et des ergothérapeutes quant aux manières d’offrir des services pour aider à renforcer les capacités des familles sans les surcharger. **Méthodologie.** Un devis descriptif qualitatif a guidé des forums communautaires en ligne avec 41 parents et ergothérapeutes du Québec, au Canada. **Résultats.** Neuf principes clés pour renforcer les capacités des familles sans les surcharger ont été identifiés. Ceux-ci comprennent : être sensibles aux répercussions potentiellement négatives des services, éviter de surcharger les familles avec des informations ou des recommandations, prendre le temps nécessaire, mettre en valeur le positif et offrir des conditions flexibles pour les services. **Conséquences.** Les résultats aident à comprendre comment les services de réadaptation en renforcement des capacités peuvent être offerts aux familles de manière à optimiser les résultats positifs et à minimiser les nuisances.

## Introduction

Occupational therapists regularly work with children who are thought to have special needs to help them fully develop their potential and participate in occupations that are meaningful to themselves and their families. A literature review by Novak and Honan (2019) indicates that the most effective occupational therapy interventions with children focus on activities chosen by the child, which offers a “just-right challenge” and are practiced in an authentic context. Parents play a key role in many of the 40 types of intervention strongly recommended by the authors, such as parental coaching and at-home activity programs. These interventions have effectively fostered the development of children's capacities and increased the confidence and competencies of parents (Graham et al., 2013; Novak & Honan, 2019). Furthermore, interventions by parents supervised by professionals and direct interventions by professionals appear equally effective (Novak & Honan, 2019). Thus, several types of intervention targeted to children or parents can generate positive benefits for the child and their family. This paper examines how interventions can build families’ capacities without overloading them.

Recognizing the crucial role of parents, King et al. (2017) conducted a literature review on rehabilitation services for families and proposed a framework of services for families in pediatric rehabilitation. It includes: (1) information or education services such as training; (2) services that equip parents to meet their child's needs such as coaching; and (3) services that meet the specific needs of parents such as support groups. Our study focuses on the first two types of services in King's and colleagues’ framework, it focuses on how occupational therapists can increase family members’ knowledge, skills, and confidence to facilitate the participation of children. It does not include interventions in which the therapist works solely with the child.

Implementing capacity-building interventions in pediatric occupational therapy calls for a major investment from parents in terms of time and travel, absences from work, or pressure resulting from the regular repetition of at-home activities. Parents of a child with special needs are already living with a high level of stress (Hsiao, 2018) and are at greater risk of difficulties related to work-family balance (Ferland, 2017; Observatoire des tout-petits, 2018). Many also experience guilt because they cannot spend enough time with their other children or take part in family activities (Myers et al., 2009; Resch et al., 2010). In this context, therapists must ensure that services offered do not place further stress on parents or complicate the establishment of a satisfying family routine.

To facilitate the engagement of parents in services, occupational therapists are advised to listen to and communicate effectively with parents, develop a trusting relationship with them, and clarify their possible role in the intervention (Novak & Honan, 2019; Phoenix et al., 2019a). In family-centered practice, therapists need to acknowledge parents’ expertise, consider the needs of all family members, and employ shared decision-making (Rodger & Keen, 2017). In a study of elements likely to impact families’ presence, participation, and engagement regarding pediatric occupational therapy services, Phoenix et al. (2019a, 2019b) underscore consideration of the family's characteristics such as the complexity of the child's state of health and that of other family members, as well as parents’ knowledge, skills, emotions, and motivation. Additionally, they pinpoint several elements related to the services themselves. These include logistical aspects, such as schedules or transportation, and the complexity of the services, including the number of organizations or professionals involved (Phoenix et al., 2019a, 2019b). Coussens et al. (2021), however, point out that professionals encounter institutional barriers when offering effective services based on the participation of children with special needs in their real-life settings. These include a predetermined number of sessions and limited access to children's life environments. Such barriers likely hinder professionals from adopting a genuinely family-centered practice.

What remains unclear is how to offer capacity-building services to families to help them foster their children's development and participation without overloading them or imposing negative effects on their satisfaction with their family routine. Although guidance on family-centered services and on how to promote the engagement of families in services is relevant, there is insufficient information on how to avoid adding pressure on families, nor specific strategies for building capacity among family members. This must be addressed to minimize families’ stress, guilt, and challenges balancing their multiple roles, including supporting their child. This study aims to describe the perspectives of parents with a child with special needs and of occupational therapists regarding promising strategies to achieve this goal of building families’ capacity.

## Method

The present study falls within the first phase of an action-research project, action planning, which is the phase prior to taking action (Chevalier et al., 2021). Action research was chosen to generate knowledge and perform actions aimed at improving services for families. It is relevant when citizens and researchers seek innovative solutions to a social problem where available scientific data are insufficient (Bradbury, 2015; Chevalier et al., 2021). The goal of the research project is to codevelop and evaluate an innovative offer of occupational therapy services aimed at building the capacities of a diversity of families having a child considered to have special needs, without overloading them. A mother of such child who works in an organization defending the rights of people with intellectual disability, and the coordinator of a social occupational therapy clinic joined the research team to share their experiential and professional knowledge. Both have actively contributed to all stages of the project and influenced decision-making since the beginning, as recommended by Palisano (2014), to help ensure findings make sense to the main people concerned by the situation. The research design for this initial phase is a descriptive qualitative study. This type of design provides a comprehensive overview of a topic from the perspectives of those who have relevant experiences (Bradshaw et al., 2017; Kim et al., 2017). We gathered perspectives of parents and occupational therapists in online community forums and in online questionnaires to provide a rich description of what can be done to build families’ capacities without overloading them. The project was approved by Comité d’éthique de la recherche sectoriel en réadaptation et intégration sociale du CIUSSS de la Capitale-Nationale (project #2021-2106, RIS).

### Participants

Participants invited to take part in online forums were required to be residents of Quebec (Canada) and belonged to two groups: parents and occupational therapists. The criteria for parents were: being the parent or a highly invested relative of a child identified as having special needs, and the child was living in the family residence with at least one parent. The criterion for occupational therapists was to work or have worked with children and their families. The choice to include therapist who had not worked directly with children and families for some time was made to enable those who were more experienced and had accepted a leadership position, as a coordinator for example, to share their ideas as well. Recruitment took place in May 2021 using convenience sampling (Fortin & Gagnon, 2016). Invitations were sent through emails and social media by the research team and organizations offering services to families (i.e., nongovernmental organizations and a health center). All participants signed an online consent form.

### Data Collection Tools

*Online community forums*. The online community forum was held twice in June 2021. Forums align well with participative processes, as they allow a large number of individuals concerned by a situation to connect, share perspectives, and find context-sensitive and sustainable solutions to a problem (Camden et al., 2014; Tétreault et al., 2013). Forums are also known to encourage original suggestions and concrete solutions for action (Camden et al., 2014; Lamontagne et al., 2014). In this project, online forums allowed parents and occupational therapists to collaborate to identify innovative strategies aimed at strengthening the capacities of families without overburdening them. These forums took place on Zoom, were conducted in French, and each lasted two and a half hours. Community forums usually include an introduction, subgroup work, and a plenary discussion (Tétreault et al., 2013). In the present case, the moderator began by presenting the schedule, research topic, and highlights of the scientific knowledge available on the issue. Participants were first divided into subgroups of three to five parents or three to five therapists for the “Sabotage” activity (Chevalier et al., 2021), which asked them to imagine the worst possible occupational therapy service for families, that is, one that not only fails to strengthen a family's capacities but also significantly overloads them as well. The main ideas were then shared at the plenary session so parents and therapists could hear the ideas of the other groups. Next, participants were divided into heterogeneous subgroups of three to five parents and therapists for the “Ideal Scenario” activity (Chevalier et al., 2021), which asked them to imagine an ideal service. The combination of these two activities aimed to stimulate the search for creative solutions while raising awareness of what should be avoided. One member of the research team was present in each subgroup to act as a moderator. This person reported the information during plenary sessions while inviting participants to clarify certain elements. Using the information provided in the sociodemographic questionnaire, the research team ensures no parents were in a subgroup with an occupational therapist they had received services from.

*Questionnaires.* Two questionnaires were developed by the research team: a sociodemographic questionnaire completed prior to participating in the forum and a post-forum questionnaire, both hosted on the LimeSurvey platform. The purpose of the sociodemographic questionnaire was to document the participants’ profile. All questions were multiple choice or short answer. The post-forum questionnaire focused on participants’ perceptions of how conducting the forum during the Covid-19 pandemic affected the exchange of ideas. Additionally, it offered an opportunity to share other ideas participants may have had after the forum. This second questionnaire, which included multiple-choice and open-ended questions, was completed by 95% of parents and 86% of occupational therapists.

### Data Analysis

Qualitative data include comments from the forum's 25 subgroups, which were recorded and transcribed in full, as well as responses to open-ended questions in the post-forum questionnaire. The analysis was structured around the following question: How can we strengthen the capacities of the family without overloading them? An inductive content analysis was performed to highlight the main themes in the body of data (Fortin & Gagnon, 2016; Patton, 2015). In line with the descriptive qualitative design, a dynamic process was followed to refine the coding tree as the analysis evolved while staying close to the participants’ language (Kim et al., 2017; Sandelowski, 2000). Three team members read the transcripts of four subgroups, then met to discuss the main themes retained and agree on a first coding tree and preliminary definitions. Next, a team member coded the data using NVivo 12 software. Another member conducted a progressive validation of the coded elements to ensure the codes’ accuracy and enable a discussion of necessary adjustments to the coding tree. A further discussion was held as needed with a third member to reach consensus. The progressive validation is intended to gather a more in-depth analysis of the data, as recommended when perspectives from discussion groups or other methods are varied and go in different directions (Patton, 2015). Given that all qualitative data were in French, the analyses were also done in French. Chosen excerpts were translated by a professional translator for the manuscript. Quantitative data from the sociodemographic information collected and post-forum questions on how the pandemic influenced the sharing of ideas were analyzed using descriptive statistics, including frequencies, percentages, and averages.

## Findings

### Participants

In total, 41 persons took part in the forums, including 19 parents (46.3%) and 22 occupational therapists (53.6%). Their sociodemographic information is presented in Table 1. All participants except for one parent identify as female (97.5%). The average age is 38 years (41 years for parents, 36 years for occupational therapists). Participants came from several regions of the province of Quebec, Canada, including cities and rural areas. Five participants (12.2%) indicated they were members of minority groups based on color of skin (*n*  =  1), immigration status (*n*  =  2), presence of a disability (*n*  =  1), or sexual or gender orientation (*n*  =  1). Regarding the parents, 84% had already received occupational therapy services; 69% in the public system, 50% in a private clinic, 12.5% in a school, and 12.5% through partnership with an association. Of note, 73.7% of parents had a university degree. The annual household income reported by parents was spread over all categories, from less than $20 000 to more than $100 000. All parents indicated that French was their first language. They had from one to four children. Most of the children considered with special needs lived with both parents (84.3%), 10.5% lived with one parent, and 5.2% lived in a blended family. These children were between 1 and 30 years old (average of 9.8 years, standard deviation: 7.09) and had a broad variety of needs and profiles (e.g., intellectual, hearing or visual disability, autism, attention, language, motor development, mental health). The occupational therapists had an average of 11.5 years of work experience (range: 0 to 29 years, standard deviation: 8.9). They worked with a wide range of clientele, in public health centers especially. Further details on their work environments and the clienteles served are presented in Table 2.

**Table 1:** Sociodemographic Information of Participants

Sociodemographic characteristics	Parents *n* (%)	Occupational therapists *n* (%)
Age		
20–29	1	7
30–39	9	8
40–49	6	4
50–59	3	3
Identification with minority groups based on:		
Color of skin	1	0
Immigration status	0	2
Presence of a disability	1	0
Sexual or gender orientation	0	1
Educational level		
Diploma of Secondary level not completed	1	0
Diploma of Vocational Studies	3	0
Diploma of College Studies	1	0
University degree (Cycle one)	10	7
University degree (Cycle two)	4	15
Annual household income		
Less than $20 000	1	NA
$20 000–$40 000	4	
$40 000–$60 000	1	
$60 000–$80 000	1	
$80 000–$100 000	3	
More than $100 000	7	
I prefer not to answer	2	
Number of children		
1	11	NA
2	4	
3	2	
4	2	
Age of their child considered to have special needs		
0–5	4	NA
6–10	9	
11–15	1	
16–20	3	
21–25	0	
26–30	2	
Profiles of their child considered to have special needs		
Intellectual disability	9	NA
Language or speech disorder	3	
Attention deficit disorder	3	
Motor challenges	3	
Autism	3	
Hearing impairment	2	
Visual impairment	1	
Challenges with sensory modulation	1	
Mental health issue	2	

**Table 2:** Work Settings and Clienteles of Occupational Therapists

Work settings of occupational therapists	*n* (%)
Work environment(s)	
Health establishment	18 (81.8%)
Private clinic	4 (18.2%)
School	3 (13.6%)
Not-for-profit social economy enterprise	1 (4.5%)
Others	2 (9.1%)
Clientele(s)	
Autism	17 (77.3%)
Attention deficit disorder	16 (72.7%)
Conduct disorder	16 (72.7%)
Intellectual disability	15 (68.2%)
General developmental delay	14 (63.6%)
Language or speech disorder	13 (59.1%)
Learning disorder	11 (50%)
Physical disability	10 (45.5%)
Mental health issue	4 (18.2%)
Orphan disease	2 (9.1%)
Chronic illness	1 (4.5%)
Hearing impairment	1 (4.5%)
Other	1 (4.5%)
Age group(s) of clientele served	
0–5 years	16 (72.7%)
6–12 years	21 (95.5%)
13–21 years	13 (59.1%)

### Ideas to Build the Capacities of Families Without Overloading Them

**Figure 1.**
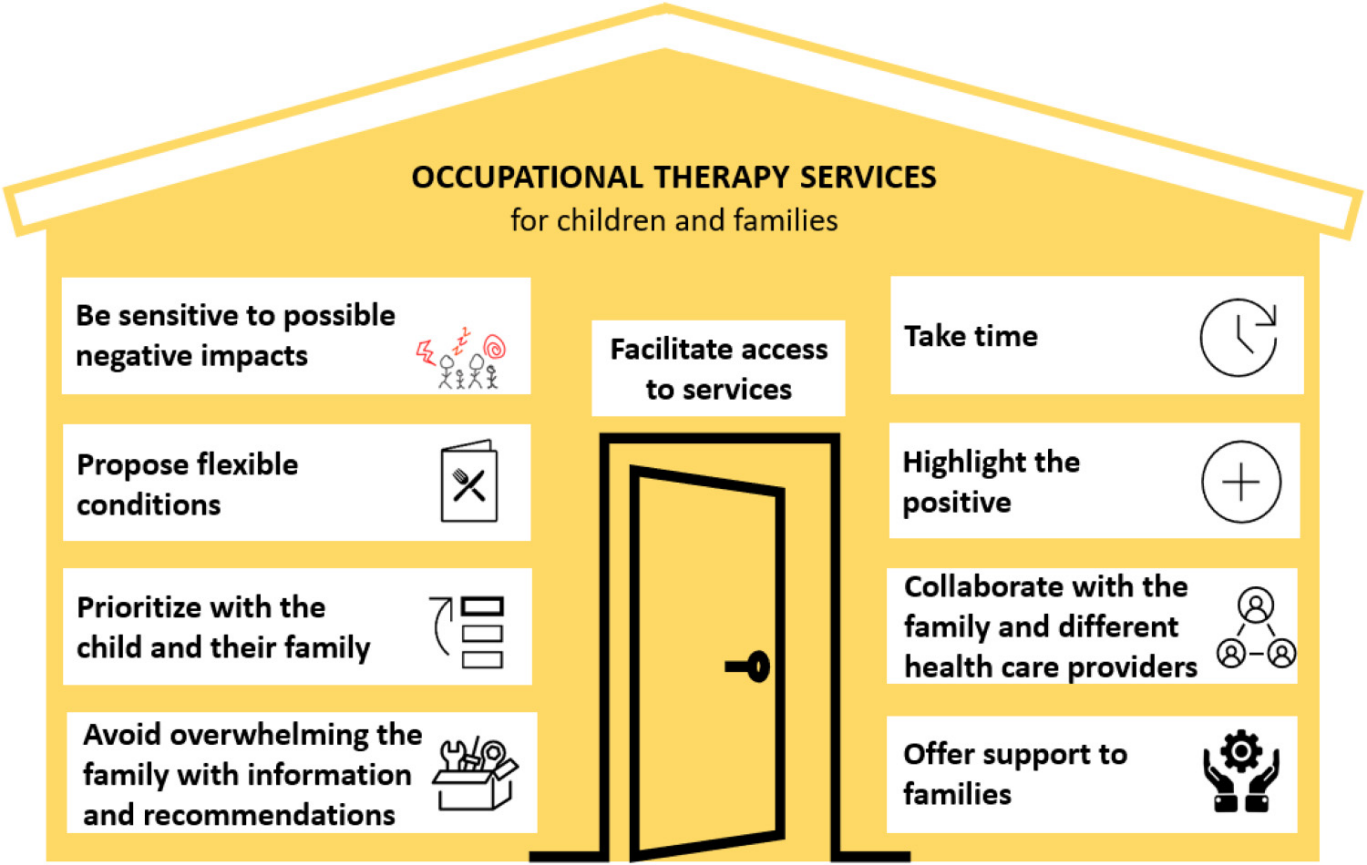
Nine key principles to build families’ capacities without overloading them.

Nine key principles were identified to strengthen the capacities of families without overloading them. These are described below and synthesized in Figure 1.

*Facilitate access to services*. To strengthen the capacities of families, participants first underscore the importance of ensuring families can access the occupational therapy services they need. Several discussed the need to access services within a reasonable time frame and avoid long waiting lists. Some participants were annoyed about the obligation to complete numerous complicated forms, which stresses the family from the start, highlighting the need to further simplify access. As well, participants emphasized the importance of ongoing access to services over a long period of time. One parent mentioned the need to feel the therapist would be “there for a little while” (F1_mixed^1^). An occupational therapist also illustrates the need to avoid service interruptions:

…for the parent has the impression they’re waiting in line to receive services and not that, each time, they change lines […] to get to the cash register, and to never feel they’re moving forward and that they have to repeat all the time. (F1_mixed)

Furthermore, both parents and therapists mentioned the importance of retaining the same healthcare providers, as much as possible, throughout the service trajectory. Finally, many participants felt that more free or low-cost services should be offered since “not all families have the means or private insurance to cover these services” (post-forum questionnaire, parent). Accordingly, parents said they wish to access more services in the public system, whether or not their child has a diagnosis and regardless of its type.

*Be sensitive to possible negative impacts*. A key theme apparent in the data is the crucial importance of therapists’ sensitivity to the possible negative impacts of their services on the child and family so ways can be found to reduce such impacts. Parents and therapists shared their concerns regarding the possible collateral effects of an intervention on parents, siblings, family life and dynamics, and relations between family members. Therapy sessions could deprive the children themselves of other important activities such as school or leisure activities. One parent gave this example: “Only right now, I have to take (child's name) out of school half a day a week so she can go to occupational therapy appointments” (F1_parent). Others mentioned the possibility that therapy sessions or at-home exercises could lead to anxiety and other negative emotions for the child.

Data also highlight possible negative impacts on parents. Some say they feel pressured to play the role of a therapist with their child, feel guilty at not doing enough, are stressed and tired, make financial sacrifices, and lack time for other occupations, including leisure activities or work, as explained by one parent: “to be able to help our child with his occupational therapy, we’d have to be at home, to not have a job” (F2_parent). Others reported that parents find it easier to do exercises or practice new tasks with the child when these activities are part of the routine, that is, when no extra work is involved. In this sense, one parent pointed to the accumulation of tasks for parents of a child with special needs to caution therapists against offering too many at-home activities:

We get overloaded real fast […] our lives are already a challenging mix of appointments, forms to fill out, requests for services that don’t exist […] anything at all [like a program of activities] to do every day isn’t realistic, even if it's two minutes. (F1_parent)

Participants also noted the importance of sensitivity to the possible impacts on other children in the family. They emphasized that the needs of siblings had to be considered. A parent who had been advised to assign responsibilities to siblings felt this was wrong: “Occupational therapists often tell me ‘oh, just ask your oldest to help you’, but [this is] not a parent, not a healthcare provider, this is his brother” (F1_parent). Finally, many participants emphasize the family's need to spend quality time together and enjoy a life outside rehabilitation services. In this regard, one parent points to the importance “of adjusting to the family's reality, like Mom who goes to work every day, or the big brother who has hockey […] so we can have quality time” (F1_mixed).

*Propose flexible conditions*. Participants explained the need for flexibility in the services offered. They cautioned against assuming the family prefers one type of session, schedule, frequency, duration, or location. A therapist illustrated this by using an analogy with fast food restaurants: “don’t use the famous Big Mac Trio […], but to offer the Subway sandwich instead, a little lettuce, 2-3 tomatoes, drop the mayo. To be able to talk about what's possible, and what's possible but not essential” (F1_mixed).

Participants made it clear that offering several ways of communication for follow-ups with parents is a winning strategy because it allows families to choose what suits them best. This may include emails or brief reports, phone calls, text messages, or videos. One parent expressed their preference as follows: “I like the phone because I can use it when moving around […] I can make calls while I’m on my way to the daycare” (F1_mixed). A therapist explained how she optimized follow-up with a parent using video capture: “[the mother] would send me a video, then I’d think ‘OK he managed that step’ […], I’d evaluate it, then I’d send a video with a strategy to practise” (F2_therapist). Regarding meeting schedules, many parents noted the difficulty of finding the right time for appointments. One, for example, stated that: “The schedule's always a problem [….]. Either we miss work, or the kids miss school, or it's after school and they’re tired or it's the weekend.” (F1_mixed). Several participants pointed out that late afternoon and early evening were difficult moments because the child is often tired and the family needs to juggle many routine tasks (e.g., dinner, homework, baths). Parents and occupational therapists also cautioned against assuming that a parent is interested in frequent sessions (e.g., weekly). Similarly, it was best not to refer to a predetermined number of sessions or duration of each meeting. Several participants brought up the importance of having the occupational therapist visit the child's different life environments, one therapist shared their view as follows: “Oh for me, my ideal occupational therapy clinic is mobile, […] having a mobile clinic would be the ideal way to meet people where they live.” (F1_mixed, therapist). Others mentioned the importance of accommodating each family's preferences and not assuming that the family wants the therapist to visit their home. Some also proposed an occasional online meeting, especially when the meeting is between the therapist and parent only.

Participants pointed to the relevance of including different people, such as parents, siblings, and relatives, in meetings with the therapist. This said, they mainly recommended flexibility regarding the attendance of each one depending on the situation. For example, a therapist indicated that at certain times, it might be convenient “to talk with only one parent, and sometimes […] for the child not to hear what the parent [is saying]” (F2_mixed). One parent suggested occasionally filming therapy sessions with the child: “these meetings […] could be filmed, because always taking a day off to see what she's doing in school, that can really be too much, but […] being able to view it could be interesting” (F1_mixed).

*Prioritize with the child and their family*. Participants stressed the importance of prioritizing objectives and interventions in collaboration with, not in place of, the child and their family so that services meet their priorities and are compatible with their preferences and culture. One therapist affirmed that: “the best service is the service adapted to the family and the child” (F1_mixed). Participants insist that objectives must address what the child and their family consider important (e.g., play with cousins), even when this is not what the therapist first had in mind. They stress this is particularly crucial when the family is from a different cultural community than the therapist. Nevertheless, some parents said they wished for some guidance. Indeed, one recounted that an occupational therapist had positively “changed the life” of their son living with developmental coordination disorder by suggesting he take up skateboarding. This parent advised that: “as a professional, sometimes you have to offer the kind of challenges that ‘shake up’ the family” (F1_mixed). Interventions should also be chosen with the family. One therapist suggests:

Having a toolbox […] that's varied enough to opt for coaching at the right time, opt for more therapies - I mean conventional therapies - at the right time, group therapy at the right time […], have as many tools as possible to properly meet the child's need […] when the child needs it, but to check with the parent that it matches the parent's needs, obviously. (F2_mixed)

Another occupational therapist noted that a therapist who develops a good relationship with the parent and takes time to understand the family's lifestyle is well placed to propose interventions or strategies better adapted to their needs. She can then suggest a few possibilities and ask them to make a choice. A parent further maintains that, although they want to help look for solutions, they also appreciate concrete suggestions from the therapist: “I expect, as a parent, to be able to propose solutions, but a lot of times I’ll say ‘I just don’t know, can you think of something? I have no idea what to do’” (F1_mixed).

*Avoid overwhelming the family with information and recommendations*. Participants emphasized that therapists should pace the information they provide to avoid overwhelming parents. They propose adapting the language used, giving just the right amount of information, and proceeding gradually. The comments of one parent are a good example: “Every time somebody gives me new things to read, to look at, I feel like, yes, I have a lot of tools, but I don’t know what to do with them because there are too many” (F1_mixed). Participants also stressed the importance of adapting the language used with the family by avoiding the use of too much theory and professional jargon.

Additionally, participants indicated that, while occupational therapists should refrain from giving too much information or too many at-home exercises, they should be open to offering more of these to parents who demonstrate interest or availability. The analysis of participants’ comments shows it's generally helpful to share the information, recommendations, or exercises given to parents gradually and to go beyond information sharing to support families in the concrete application of strategies. This can be done by practicing the strategies with the family or helping parents implement the recommendations in their everyday life.

*Take time*. Several participants pointed out the importance of taking time to forge a relationship with the child and the family, explain interventions, and conduct follow-ups. To illustrate the necessity of forming a relationship, one parent stated: “there's also the human aspect, I wouldn’t want a therapist who's kind of a robot” (F1_mixed). Several therapists and parents indicated that it is essential to take time to explain to parents the reasons for occupational therapy as well as the recommendations and how they can be applied in their child's daily life. One parent commented: “I need to understand so I’ll be motivated to apply it so it makes sense to me” (F2_parent). Some parents also mentioned they wanted therapists to take time to answer their questions.

A number of parents said they appreciate the therapist's taking time to conduct frequent follow-ups with them. Although therapists were open to this suggestion, some said they feel their practice does not allow enough time for this. Others feel that not all families necessarily have time for this type of follow-up, again underscoring the importance of adapting to each family's needs and reality. One therapist stated that “[it's not] that the parent [isn’t] motivated, it's that he is already overloaded in his daily life […] some families are not going to call me back, it will wait till the next appointment” (F2_mixed).

*Highlight the positive*. Some participants underscore the importance of highlighting the positive in interactions with children and parents. One parent explained that “our children experience a lot of failures in their everyday life” (F1_mixed). Highlighting the positive includes pointing out the child's strengths, regularly congratulating the parents and child for their efforts, and emphasizing their progress or “small victories.” In the words of one parent:

It's so slow [before seeing progress], that we [don’t] notice if he's improving because we’re there every day […] we get the feeling it's useless, […] telling us “well look, he's moving his tongue differently,” the occupational therapists […] they notice things. (F1_parent)

*Collaborate with the family and different health care providers*. Participants emphasized the value of collaboration with the different persons involved in the child's life. They insisted that the therapist's collaboration with the family, particularly how they communicate with them, is an essential factor in services aimed at building families’ capacities. Parents explained that they know their child very well and can therefore help the therapist understand the child's particular situation when their input is sought and appreciated. Certain participants also suggested the occupational therapist collaborate with the different professionals involved with the child—so the parent can avoid starting over with each one and ensure the family is offered complementary and realistic services.

To this end, a number of participants, including the parent quoted below, proposed the equivalent of a “multidisciplinary meeting with the parent that will be attended by all professionals accompanying the child” (F2_mixed). The aim would be to coordinate efforts around priorities established as a team. Certain participants also suggested a patient navigator be named to facilitate collaboration and to prevent a parent from taking on this role.

*Offer support to families*. The last key message groups the different forms of support available to help families actively engage in services, namely, in-home support by trained workers, support from relatives, and help accessing required equipment. The contribution of occupational therapy students, rehabilitation technicians, or educators was proposed to assist families to implement recommendations or activities at home. One parent shared their vision:

A person who’d help us at home who’d be, let's say, a ‘wannabe occupational therapist’, either an occupational therapy student, or intern or assistant educator […] I [wouldn’t] pay a professional salary, but I’d pay them pretty well as a babysitter to help us do all the exercises. (F1_mixed)

Support from relatives could also be a way to lighten the load, as parents propose that they be called on to perform the recommended activities with the child.

Finally, many families worry about the cost and availability of the equipment needed for their child. Participants argue that therapists should consider less expensive alternatives to specialized equipment and explore those that are already available and easy to use. One parent proposed “maybe going [as far as] the loan of equipment, […] equipment for children who are different is quite expensive, so [we should] be able to access it more easily at a lower cost” (F1_mixed). Therapists brought up the need for time to create equipment geared toward families—stimulation boxes, for example—and to research nonspecialized equipment that could also help the child and family achieve their objectives.

### Impact of Pandemic on Shared Ideas

In the post-forum questionnaire, 43% of participants said they thought the pandemic influenced shared ideas during the forums. They noted that they gained more experience with online meetings during this time. Therapists pointed out that such meetings offered further possibilities for therapy services, while some parents said they did not appreciate their experiences with telepractice or online schooling for their younger children. A few also mentioned they were overburdened during the pandemic even before trying to integrate occupational therapy into their daily routine. This situation appeared to encourage some professionals to refocus their practice on the most important aspects of the family's daily life. Participants also noted that the reduction in rehabilitation services during the pandemic further demonstrated the importance of access to these services.

## Discussion

This study identified potential solutions for providing occupational therapy services that help strengthen the capacities of families with a child considered to have special needs and to do so without overburdening them. The importance of considering the perspectives of the child and their family in key decisions concerning services was emphasized above all. Many other studies likewise stress the importance of using the expertise of the child and their family; supporting them to make choices based on their values, interests, and needs; and enabling their participation in the search for solutions (Coussens et al., 2021; D’Arrigo et al., 2020; Fragasso & Pomey, 2021; Palisano et al., 2012; Palisano, 2014). Our study adds to the knowledge base on how to involve families in decisions regarding the type of interventions offered (e.g., coaching, educational videos, groups) and service modalities (e.g., videoconference, location of the services, frequency). Forum participants stressed that it is essential not to assume the child and family share the same vision as the professional in terms of objectives. This is congruent with a call from other authors (Cramm, 2011; Rosenbaum & Gorter, 2011) to move away from what is expected at a certain age in a typical development, or from how things should be done, to focus on what really counts for children and families, helping them do what they wish to do in a manner that is meaningful for them. Coaching approaches appear particularly promising to focus on what is important for children and families because parents become better able to meet their child's needs and strengthen their sense of self-efficacy through coaching (Fragasso & Pomey, 2021). Occupational performance coaching, for example, employs collaborative performance analysis of how the child performs in their chosen occupations to support the identification of realistic strategies to implement (Graham et al., 2020). This approach is recognized as effective for improving children's participation and parents’ satisfaction with chosen occupational objectives (Graham et al., 2013; Novak & Honan, 2019).

This study's findings underscore the importance of increasing rehabilitation professionals’ awareness of the possible negative impacts of services on children and families. This can include parents feeling guilty or feeling under too much pressure. This echoes Courcy's (2013) indications that, for some parents, assuming the role of therapist with their child, when performing exercises at home, for example, may lead to stress and fatigue. Our findings also highlighted that therapy or exercises done at home can mean that the child or his parent will miss important activities and can provoke negative emotions as the child might feel incompetent, not good enough. Similarly, Cramm (2011) warned therapists that they might have detrimental impacts on children's mental health if they convey a message that children should be developing in a certain way and should work hard to meet the norms. Our findings also put forward that families thought that spending quality time together was critical and that therapy (e.g., meetings and home programs) can sometimes hinder them from achieving this. Professionals must, therefore, be aware of the risk of causing harm to the child and his family and be ready to discuss this sensitive topic openly with them, making sure they really build capacity in a manner that does not overload families or make them feel bad about themselves.

The offer of flexible conditions and means of communication emerges as another essential factor in building the capacities of families. This is not surprising since the rigidity of services is a major challenge for many families (Roy et al., 2021). Flexibility requires therapists to take into account the work commitments of parents, the schedule of different services received simultaneously, and transportation issues (Phoenix et al., 2019a; Rodger & Keen, 2017). This can help limit the harm done by the therapy, if one is mindful of the important activities for the child and their family and if the conditions in which the services are offered are tailored to the families’ needs as much as possible.

Whereas our results underscore the importance of quick and easy access to services, flexible conditions, and professionals who take time with each family, in certain places like the province of Quebec, Canada, long waiting lists or insufficient resources in the public health system impose organizational constraints on professionals and can cause many families to turn to private clinics (Observatoire des tout-petits, 2021). This situation risks placing financial pressure on families and accentuating inequalities among children based on their parents’ ability to pay. Some characteristics, including low socioeconomic status and ethnic minority, place some people at higher risk of encountering barriers to health care (Fujiwara et al., 2022; Salloum et al., 2016; Shenouda et al., 2022). Although children and families with the greatest need for services are known to encounter the most structural barriers to accessibility (Human Early Learning Partnership, 2013; Institut national de santé publique du Québec, 2014), it is crucial to facilitate access to services at the right time for all families with needs if we are to really build their capacities without overloading them.

Yet, when resources are limited, making sure that all families, including those with the greatest need, access services that respond to their needs quickly is a challenge. Building on recommendations from other authors (Coussens et al., 2021; Cramm, 2011; Palisano et al., 2012; Rosembaum & Gorter, 2011), our findings call for a change in philosophy regarding services for children and their family to help them with what they think is important in a manner that is respectful of who they are and their daily routine. We must depart from the usual solution of proposing individual sessions focused on children's skills development so that they can meet the norms for typical development (Cramm, 2011; Rosembaum & Gorter, 2011). We must rather focus on strengthening the capacities of children, parents, and communities to identify and implement their own solutions when faced with a challenge (Coussens et al., 2021; Palisano et al., 2012; Rosembaum & Gorter, 2011). In other words, it is time to focus on building the capacity of the people who are with the children everyday so that they can help the children do what they wish to do in a way that makes sense for them. Multitiered service delivery (Bazyk, 2011) and local services offered in partnership with the organizations and settings where children and families evolve (Roy et al., 2021) should be considered. A service offer of this kind supposes that occupational therapists provide: (1) universal services targeting all children and the key actors in their environment; (2) services targeting children presenting challenges despite the universal services deployed; and (3) more intensive services adapted to the needs of children with ongoing challenges. Although this type of approach is recommended and has been effective in school settings (Bazyk et al., 2018; Missiuna et al., 2015), it is rarely used in other contexts and has received little research attention. Accordingly, this would be worth exploring in both research and clinical services in occupational therapy.

Even if multitiered services were to be offered, one must ensure that the children and the families with the greatest need really access them. Conceiving access to services based on targeted universalism (Powell et al., 2019) is another promising avenue. This approach recognizes that certain groups within that population face structural barriers to access services. Targeted strategies are then implemented to overcome these barriers to facilitate access for a greater diversity of families. Community Outreach is an example of such strategy. Roy et al. (2021) demonstrated that the presence of a community worker in a disadvantaged area helped families develop greater trust and confidence in the services available for their children. Other examples include paying attention to the language in which the services are offered, the location where they are offered as well as the knowledge and perspectives held by different underserved communities regarding the services available.

### Strengths and Limitations

The study is rooted in a methodology where scientific, professional, and experiential knowledge combines to develop creative solutions to challenges regarding the offer of services to children and their families. The involvement of two persons with experiential and professional knowledge in the various stages of the research project has helped align the project with actual needs and promote scientific culture in the two organizations they represent. The perspectives of occupational therapists and parents were shared during the forums. All but one participant reported feeling comfortable with sharing their ideas and appreciated hearing the perspectives of the other participants all the time or the majority of the time. Yet, one parent and one occupational therapist reported finding it more difficult to express their ideas in the mixed subgroups activity. The convenience sample allowed for recruiting participants from different regions, parents of children whose needs were associated with various conditions, and occupational therapists working with various clienteles in the public system, private practice, and not-for-profit organizations. The research team, however, recognizes the relative homogeneity of the parents who participated in terms of gender and cultural groups. It is, therefore, possible that the key principles identified do not meet the needs of fathers or families from other cultural backgrounds than participants. Collaboration with organizations offering services to different cultural communities can address this in future studies.

## Conclusion

The principles presented here contribute to the advancement of knowledge on capacity-building rehabilitation services for children and their families, by clarifying ways to offer such services without overloading the families. Notably, study participants stress the need for professionals to be sensitive to the possible negative impacts of their services and to avoid making assumptions about what children and families need. Occupational therapists and other rehabilitation professionals offering services to children and their families can apply the principles identified in this study, reflect on their practice, and make changes if needed. Another hope is that public health services will be able to offer rapid, ongoing access to human and flexible services that meet the needs of all families who need them. The next phase of this action-research project consists of accompanying occupational therapists in attempting to apply these principles in real life with five to ten families while documenting challenges, potential solutions, and outcomes.

### Key Messages

- Occupational therapists need to be aware of the possible negative impacts of their services on children and their families, even when the therapist is trying to help.
- It is essential to focus on families’ most pressing needs and to offer flexible conditions as no one-size-fits-all services can build all families’ capacities without overloading them.
- Health services administrators should be made aware of the conditions in which capacity-building services should be offered to help families support their child considered to have special needs without overloading them.

## Notes

1. Sources of citations in this article are indicated as follows: F1 (forum 1), F2 (forum 2), mixed (subgroup with parents and occupational therapists), parent (parent subgroup), and therapist (occupational therapist subgroup). The text specifies if the comments were made by a parent or therapist.
